# Simulation in podiatry teaching and learning: A scoping review

**DOI:** 10.1002/jfa2.70020

**Published:** 2024-12-03

**Authors:** Naomi Anning, Peta Tehan

**Affiliations:** ^1^ Department of Vascular Surgery Sunshine Coast University Hospital Birtinya Queensland Australia; ^2^ School of Medicine and Public Health University of Newcastle Newcastle New South Wales Australia; ^3^ Faculty of Medicine Nursing and Health Sciences Subfaculty of Clinical and Molecular Sciences Monash University Melbourne Victoria Australia; ^4^ School of Health Sciences College of Health Medicine and Wellbeing University of Newcastle Newcastle New South Wales Australia

**Keywords:** learning, medical education, podiatry simulation, scoping review, teaching

## Abstract

**Background:**

In podiatry, there are a variety of clinical tasks that require precision and skill and it is expected that clinicians will obtain these skills during their training. Simulation is a dynamic teaching tool used in healthcare to enhance skill and knowledge acquisition. Currently, the extent and nature of the research on the use of simulation in podiatry teaching and learning are not clear.

**Aim:**

A scoping review was conducted to identify the extent and nature of research activity on the use of simulation in podiatry teaching and learning and identify gaps in the existing literature.

**Methods:**

Any research relating to simulation use in podiatry teaching including various designs and focusing on simulations aimed at improving podiatry teaching or learning were eligible for inclusion. A systematic search was conducted on February 14, 2024 of the following databases: Embase (via Embase.com), MEDLINE (via PubMed), CINAHL, and the Web of Science. Additional papers were identified via bibliographies of included studies. Content analysis of content relating to podiatry teaching and learning was performed and grouped into broad themes, then further narrowing to six themes.

**Results:**

A total of 21 research studies were deemed eligible for inclusion focusing on diverse aspects of podiatry simulation utilized in high‐income countries exclusively. Conducted between 1997 and 2023, these studies were categorized into six key themes: skill improvement, communication and professionalism, clinical competencies and patient safety, educational enhancement, and anatomy and histology education. The simulations, carried out by or assessed for podiatry professionals, staff, or students, ranged from high‐fidelity medical mannequins to low‐fidelity simulations such as a grapefruit model of a diabetes‐related foot ulcer.

**Conclusion:**

Overall, the findings suggest that simulation teaching in podiatry, whether through direct skill enhancement or through educational impact assessments, holds potential in improving competency, confidence, and educational outcomes in podiatry practice. This scoping review identified a small yet diverse evidence base for simulation modalities in podiatry education, demonstrating gaps in long‐term effects and comparative effectiveness studies. It highlights the urgent need for research focused on longitudinal impacts, evaluating various simulation technologies and standardizing best practices to improve podiatry education and align with clinical and patient care needs.

## 1 INTRODUCTION

Simulation in healthcare is a dynamic teaching tool that serves to enhance skill and knowledge acquisition in various clinical scenarios [1, 2]. It is defined across a spectrum from high‐fidelity simulations, which recreate complex clinical environments using sophisticated mannequins and computer‐based systems to low‐fidelity simulations, which utilize simpler inanimate simulators for basic procedural training [1, 2]. Simulation also encompasses the use of animal and human cadavers, offering realistic anatomy and haptics for surgical training, though ethical and cost considerations have impacted their popularity [1]. Simulated patients, or standardized patients, represent another facet of simulation where individuals are trained to act out scenarios in a consistent manner allowing for the practice of communication skills, history taking, and physical examination in a safe and controlled setting [3]. Additionally, virtual reality (VR) simulations provide immersive interactive learning experiences though they may be cost‐prohibitive [1, 2]. This educational approach allows for the safe practice of clinical skills without the risk of harming actual patients making it an invaluable component of health professional training [1, 2].

Simulation finds its application both in the educational domain for teaching and learning, such as in the undergraduate and postgraduate medical training and education [4, 5], but also in clinical settings to enhance patient care outcomes, for example, to increase patient safety and quality improvement [6, 7, 8, 9] or improve patient education and empowerment [10]. In clinical teaching, simulation is a vital tool for developing and evaluating key clinical competencies, such as procedural skills and clinical reasoning, providing a risk‐free environment for repetitive practice [11, 12]. It addresses limited patient availability and the necessity for reliable competence assessment [12], playing a crucial role in securing healthcare professionals' competence and boosting patient safety by potentially reducing medical errors [13, 14, 15].

Simulation‐based medical education enhances healthcare teaching and learning by facilitating deliberate clinical skill practice, boosting knowledge retention, and confidence [4, 16], however further evidence of its clinical effectiveness is needed [17]. Despite its cost and complexity, simulation is becoming integral to health professional curricula [18], especially high‐fidelity simulations in nursing and allied health [19]. It has potential to improve clinical competency, patient safety, and accommodates growing student numbers [20, 21], with effective feedback and debriefing being crucial [22]. Simulation notably enhances students' knowledge, skills, and self‐confidence [23].

Podiatry practice contains a variety of skills and knowledge tasks that require the practitioner to apply high levels of precise manual skills [24]. Furthermore, podiatrists require high levels of clinical reasoning as independent practitioners [25, 26]. The use of simulation in podiatry education potentially offers immersive learning for mastering foot and ankle anatomy, safe surgical practice on high‐fidelity models, and development of decision‐making skills through clinical scenarios [27, 28, 29]. It may be able to foster interprofessional collaboration, enhance teamwork, and build student confidence providing customizable and feedback‐rich learning experiences for a competent start in podiatry [29, 30].

However, to date, the nature and extent of research regarding podiatry simulation in teaching and learning have not been comprehensively mapped, with no systematic reviews or comprehensive syntheses of evidence available in the literature. Therefore, the aim of this scoping review was to map the current extent and nature of research activity on the use of simulation in teaching and learning in podiatry and identify areas for future research.

## 2 METHODS

The methodology of the scoping review was selected for its efficiency in quickly identifying essential concepts, sources, and evidence types especially useful in areas that are complex or have not been extensively explored before [31]. This approach utilized the Arksey and O'Malley framework, further adapted by Levac [31, 32], excluding the consultation phase. The presentation of findings adhered to the PRISMA‐ScR guidelines [33].

### 2.1 Eligibility criteria

The inclusion criteria were kept broad to include as much as available research on the topic.

Any type of study design was accepted including experimental and quasi‐experimental studies, qualitative studies, observational studies, and cross‐sectional studies as well as reviews and expert opinions. The intervention assessed, described, reviewed, or investigated must have been any type of simulation with the aim of enhancing teaching or learning podiatry skills, carried out by podiatry professionals or students, and/or assessed by podiatry faculty staff. Studies where a mixture of podiatry staff/students was involved together with other medical professions were also included. No restrictions on types or presence of comparators and outcomes were made.

No time limitation for the publication date was set. The search was limited to the English language.

The following studies were excluded.

Studies of which no full‐text was available (abstract only), studies in languages other than English (language), and studies investigating educational or teaching simulations directed at professionals or students from other disciplines than podiatry (intervention).

### 2.2 Information sources, database search, and study selection

MEDLINE (via PubMed), Embase via Embase.com, CINAHL, and the Web of Science were searched on February 14, 2024.

The search strategies were adapted for each database; the following search strategy was used for MEDLINE: podiatr* AND (virtual OR simulat* OR cadaver) and adjusted appropriately for subsequent databases.

During the initial phase of selection, two separate reviewers (NA and PT) independently examined the titles and abstracts of all gathered studies to determine their suitability according to the inclusion criteria. Subsequently, the complete texts of the preliminarily selected articles were independently evaluated by two reviewers (NA and PT) to decide their final inclusion in the study. Any arising disagreements were settled through discussion. The bibliographies of the reviews and articles that were acquired were also reviewed to identify any additional studies for inclusion. In cases where further details about the study were required, the authors of the publications were contacted. Additionally, searches for 'grey literature,' such as Doctoral and Master's theses, were conducted using Google Scholar.

### 2.3 Data extraction

The process of data extraction was carried out manually by a single reviewer (NA) into an Excel spreadsheet and was subsequently verified by another reviewer (PT). The data elements gathered included: author, publication year, country, type of participants, study design, aim of study, simulation modality, purpose of the simulation, and the main findings of the research study.

### 2.4 Data charting and items

A content analysis was performed, and data were organized into tables by purpose of simulation intervention: skill improvement, communication and professionalism, clinical competencies and patient safety, educational enhancement, and anatomy and histology education. Data were further described according to study design, participants, overall study aim, simulation modality, and main findings. The aim of this structure was to efficiently summarize the literature so as to draw links among the purpose of the simulation, its modality, and the main findings. Further quantitative synthesis was not possible due to the diverse scope of included data.

## 3 RESULTS

### 3.1 Study searches and selection

**FIGURE 1:**
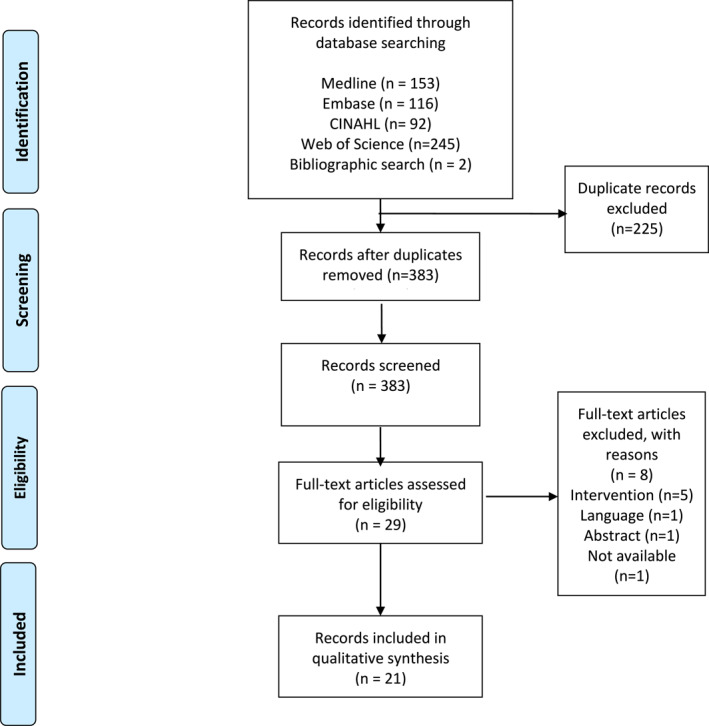
PRISMA flow diagram.

The comprehensive search of databases resulted in 608 entries. Following the elimination of duplicates and the exclusion of studies based on the eligibility criteria, 29 full‐text articles were evaluated for their potential inclusion in this review. With the removal of 8 articles, 21 studies were ultimately selected for inclusion in this review (Figure 1).

### 3.2 Study characteristics

There were a diverse range of study characteristics across the included papers. The years of publication ranged from 1997 to 2023, showcasing a broad temporal scope of research in this field. From the reviewed studies, a notable geographical distribution in high‐income countries was observed with studies conducted in the USA (*n* = 11), Australia (*n* = 6), the United Kingdom (*n* = 3), and Finland (*n* = 1) reflecting a significant interest in podiatry simulations across these countries (Table 1). The study designs utilized in the podiatry simulation research literature varied widely including experimental studies (*n* = 1), quasi‐experimental studies (*n* = 5), surveys (*n* = 8), mixed methods studies (*n* = 2), qualitative studies (*n* = 2), laboratory studies (*n* = 1), and narrative reviews (*n* = 2) (Table 1).

**TABLE 1:** Characteristics of the included studies.

Study	Country	Participants	Study type	Study design	Overarching aim of study	Simulation modality	Purpose of simulation	Main findings
*Skill improvement*								
[34]	Finland	Graduating student nurses, graduating student podiatrists, registered nurses, and podiatrists	**Quasi‐experimental study**	Interventional, no control	To describe the level of chronic wound‐care competence among graduating students compared with professionals and to develop and test the C/WoundComp instrument	Practical examination using an anatomic wound model to simulate a diabetic foot ulcer	Assess practical wound‐care competence	The students' total mean competence score was 62%, with a theoretical competence mean score of 67% and a practical competence mean score of 52%
								Students' competence level was significantly lower than that of professionals, but students showed a positive attitude toward chronic wound care
								The developed instrument, C/WoundComp, demonstrated preliminary validity and reliability for assessing wound‐care competence
								Significant differences in competence levels were observed between students and professionals, with professionals demonstrating higher competence in both theoretical knowledge and practical skills
[35]	Australia	Undergraduate podiatry students	**Mixed methods study**	Repeat measure, RCT, and focus group	To study the effects of simulation in terms of anxiety levels, confidence, and fidelity	3D printed foot model, foot ulcer models	Practice scalpel skills	RCT: In comparison to standard teaching methods, the use of 3D printed foot models had similar decreases in anxiety and increases in confidence measures
								Repeat measure study: The use of 3D printed foot models increased both novice and more experienced users' self‐confidence and task self‐efficacy
								Focus group: Students identified the use of 3D foot models for the learning of scalpel skills as ‘authentic’ and ‘lifelike’
[36]	USA	Podiatric residents	**Cross‐sectional study**	Survey	Assess cadaver lab structure and availability in podiatric surgical training programs	Cadaver labs, inanimate simulators, and animal models	Hands‐on surgical training and technique familiarization	Majority found cadaver labs beneficial
								Key factors: faculty instruction, lab accessibility, and instrumentation availability
								High value for surgical technique and anatomy familiarization
[29]	Australia	Podiatry students	**Experimental study**	Pilot‐RCT	To study the effects of simulation (simulation plus clinical placement vs. clinical placement alone)	Simulation workshop of a medical foot‐care model for sharp debridement practice	Improve debridement skills and confidence	Intervention group 16 times more likely to be assessed as competent in debridement skills
								Intervention group reported increased confidence in skills compared with the control group
[38]	UK	Newly qualified podiatrists	**Quasi‐experimental study**	Interventional, no control	Train podiatrists in focused duplex ultrasound scanning of tibial vessels at the ankle in diabetic patients (PodAnk duplex scan)	High‐fidelity pulsatile flow simulator with a straight vessel phantom	**Teach and assess PodAnk duplex scanning proficiency**	High accuracy in detecting the loss of triphasic flow (92.9%) and correct waveform (89.1%); excellent imaging scores indicating proficiency; mean time for bilateral ankle assessment: 20.4 min
[39]	Australia	University podiatry program staff	**Qualitative study**	Interview study	To explore scalpel skill teaching approaches, issues, and innovations in podiatry programs in Australia and New Zealand	Low‐fidelity simulations using inanimate objects (i.e., oranges, avocadoes, and impregnated materials)	Practice scalpel skills	Teaching approaches were relatively consistent across programs, involving didactic content, demonstration, practice on inanimate objects, and supplementary materials
								Main issues included student anxiety, motivation, and dexterity challenges
								Successes included the use of low‐fidelity simulation for initial practice and structured progression to more complex tasks
								Strategies for managing poor performers included increased teaching exposure, positive reinforcement, and simplifying tasks
[40]	UK	Podiatrists	**Mixed methods study**	Descriptive method development, survey	Develop a model for sharp debridement of DFUs for podiatry training	Grapefruit model with irregular‐shaped ulcer simulation	Practical training tool for sharp debridement of DFUs	The grapefruit model was found to be an easy‐to‐understand, suitable training tool for DFU debridement, rated with an average utility score of 61.9% by podiatrists
								The model was inexpensive, accessible, and supported by freely available software for assessing debrided area and depth
[37]	USA	Not specified for direct involvement; method focused on podiatry students and residents' education	**Laboratory study**	Descriptive method development	Improve anatomic identification in podiatric education using a novel dissection technique	Cadaveric foot and ankle structures	Enhance learning and preservation of anatomic structures	Improved preservation and identification of anatomic structures
								Method required additional preparation time but was beneficial for educational purposes
[41]	Australia	Base level ‘clinician’ (Level 3) or ‘senior clinician’ (Level 4) podiatrists	**Quasi‐experimental study**	Before–after study	To evaluate the effectiveness of a 2‐day practical simulation training workshop (FUST)	Repetitive practice at part task "stations," scenario‐based simulations with foot models, and simulated clinical outpatient environments by standardized patients (actors) with simulated foot ulcers	Enhance clinical skills and decision‐making	FUST program was effective in increasing podiatrists' confidence in managing foot ulcers, with significant improvements observed in both theoretical knowledge and practical skills as assessed through simulation‐based training
*Communication and Professionalism*								
[42]	Australia	Facilitators (amongst them one podiatrist), 12 simulated clients, and 40 students (amongst them a few podiatry students)	**Qualitative study**	Qualitative evaluation	Explore stakeholders' perceptions of training for and conduct of simulated client‐based activity	Simulated client interprofessional Education (SCIPE) program	Support the development of collaborative practice	Improvements in students' communication and awareness of interprofessional collaboration
[43]	USA	Second‐year podiatric medicine students	**Quasi‐experimental study**	Interventional, no control	To examine differences between faculty and SP evaluations on student professionalism during a simulated encounter	Simulated patients with peripheral vascular disease	Teach and assess professionalism and communication	Significant differences in "build a relationship," "gather information," and "share information" domains between faculty and SP scores
[30]	USA	Podiatric medical students and nurse anesthesia students	**Quasi‐experimental study**	Befor–eafter study	Determine the impact of interprofessional podiatric surgical simulation on perceptions of the students	Hypothetical patient case study involving an infected diabetic foot ulcer	Influence interprofessional perceptions	Statistically significant differences observed in perceptions before and after the simulation
*Clinical Competencies and Patient Safety*								
[44]	USA	Podiatric colleges in the US	**Cross‐sectional study**	Survey	Assess the use of simulation‐based education in colleges of Podiatric Medicine in the US	Any	Teach and assess clinical competencies	In all participating schools, simulation was used in education and teaching although the use varied
[45]	USA	US schools of podiatric medicine	**Cross‐sectional study**	Survey	To describe the current state of emergency medical training in US schools of podiatric medicine and to encourage further studies on the effectiveness of simulation in the podiatric medical curriculum	High‐fidelity simulation	Teaching the Management of Medical emergencies	All US schools of podiatric medicine integrate emergency medical training into their curriculum, employing both didactic lectures and clinical hands‐on components, including simulation
[27]	USA	Third‐year podiatric medical students	**Cross‐sectional study**	Survey	To assess podiatric medical students' perceptions of simulator use after a third‐year simulation rotation	High‐fidelity medical mannequins used in a simulation laboratory as a part of the podiatric medical curriculum	Enhance clinical skills and patient safety	Podiatric medical students perceived simulation as an effective educational tool
[46]	USA	Not specified for direct involvement; podiatric medical education	**Review**	Narrative review	Focus on the use of patient simulators in podiatric medical/surgical education	Patient simulators	Teach and assess clinical competencies	N/A
*Educational Enhancement*								
[47]	Australia	Allied health students from disciplines of occupational therapy, physiotherapy, podiatry, and speech–language pathology at a regional Australian university	**Cross‐sectional study**	Survey	To explore the perspectives, learnings, and experiences of allied health students who participated in a large, multicampus, and interprofessional clinical simulation experience	Clinical simulation involving cross‐disciplinary activities designed to reflect real‐world practice	Provide authentic educational opportunities for interprofessional practice	Allied health students valued the interprofessional simulation for enhancing their understanding of other professions, collaboration importance, and communication confidence, despite logistical challenges across campuses
[48]	USA	Osteopathic and podiatric medical students	**Cross‐sectional study**	Survey	To understand students' perceptions of integrating high‐fidelity simulation exercises into human physiology curricula	High‐fidelity simulation scenarios related to pulmonary physiology (pneumothorax case) and gastrointestinal physiology (gastrointestinal bleed case)	Reinforce physiological concepts; enhance understanding	Students across both osteopathic and podiatric medical programs found high‐fidelity simulation exercises beneficial for understanding human physiology concepts within a clinical context; osteopathic students perceived these simulations as more valuable
[28]	USA	Not specified for direct involvement; podiatric medical education	**Review**	Narrative review	Focus on the role of VR simulations in podiatric medicine	VR simulations	Improve teaching and learning	N/A
*Anatomy and Histology Education*								
[49]	USA	Podiatric Medical and pathology Assistant students	**Cross‐sectional study**	Survey	To study attitudes toward simulation	Virtual microscopes	Teaching tool for histology education	Majority of students viewed virtual microscopy positively for enhancing their understanding of histology
[50]	UK	First‐year podiatry students at the University of Edinburgh	**Cross‐sectional study**	Survey	To determine the reactions of podiatry students to their initial experiences in the anatomy dissecting laboratory	Human cadaver dissection lab	Introduce podiatry students to structural anatomy	Students felt unprepared for cadaver dissection, experiencing significant negative reactions, and suggested the need for better preparatory and postexperience support

Abbreviations: DFU, diabetic foot ulcer; DOPS, direct observation of procedural skills; FUST, foot ulcer simulation training; N/A, not applicable; PAD, peripheral artery disease; RCT, randomized controlled trial; SP, standardized patient; UK, United Kingdom; USA/US, United States of America; VR, virtual reality.

The identified studies addressed different aspects of simulation in podiatry education with distinct simulation purposes, illuminating the multifaceted applications and outcomes of simulation‐based education across various domains, and emphasizing skill improvement, communication and professionalism, clinical competencies and patient safety, educational enhancement, and anatomy and histology education (Table 1).

#### 3.2.1 Skill improvements

Kielo‐Viljamaa et al. [34] and Banwell et al. [35] aimed to improve wound care and scalpel skills using anatomic and 3D‐printed models. Chu et al. [36] and DiLandro et al. [37] enhanced surgical skills and anatomic knowledge through cadaver labs and innovative dissection methods. Grollo et al. [29] and Normahani et al. [38] focused on procedural skills such as debridement and duplex ultrasound scanning, respectively, demonstrating significant advancements in technical skills and diagnostic capabilities. The studies by Causby et al. [39] and Jackson et al. [40] employed low‐fidelity simulations for teaching scalpel and sharp debridement skills underscoring the effectiveness of simple models in skill acquisition. Lazzarini et al. [41] evaluated a comprehensive training workshop that led to notable improvements in clinical skills and decision‐making (Table 1).

#### 3.2.2 Communication and professionalism

In the domain of communication and professionalism within podiatry education, Waller and Nestel [42] utilized the Simulated Client Interprofessional Education program to significantly enhance students' communication abilities and interprofessional awareness, whereas Mahoney et al. [43] uncovered notable discrepancies between faculty and standardized patient assessments of student professionalism, particularly in building relationships and sharing information. Meanwhile, Mendel et al. [30] observed a marked improvement in students' perceptions of interprofessional collaboration following a podiatric surgical simulation indicating the potential of simulation to cultivate essential professional competencies (Table 1).

#### 3.2.3 Clinical competencies and patient safety

In the sphere of clinical competencies and patient safety in podiatry education, Errichetti et al. [44] surveyed simulation‐based education's broad but varied use in U.S. podiatric colleges, whereas Wald et al. [45] detailed the integration of high‐fidelity simulations for emergency medical training in podiatric curricula, stressing its effectiveness in clinical emergency management. Smith et al. [27] examined podiatric medical students' perceptions of high‐fidelity mannequins for improving clinical skills and patient safety. Muscarella [46], although not providing specific outcomes, focused on the use of patient simulators in podiatric medical and surgical education, suggesting an underlying recognition of simulation's potential to enhance clinical competencies (Table 1).

#### 3.2.4 Educational enhancement

In the field of educational enhancement within podiatry education, Robson et al. [47] highlighted the positive impact of interprofessional clinical simulations across multiple campuses on students' understanding and skills in collaboration and communication despite logistical hurdles. Olson et al. [48] demonstrated the effectiveness of high‐fidelity simulations in human physiology curricula for osteopathic and podiatry students, emphasizing their role in deepening physiological knowledge and clinical understanding, with osteopathic students valuing these experiences highly. Labovitz and Hubbard [28] discussed the promising potential of VR simulations in podiatry, suggesting their capacity to advance teaching and learning, though detailed outcomes were not specified (Table 1).

#### 3.2.5 Anatomy and histology education

Becker [49] and Weir and Thomas [50] explored the use of virtual microscopes and reactions to cadaver dissection in podiatry education, respectively. Becker found positive student perceptions toward virtual microscopy for histology, whereas Weir highlighted students' unpreparedness and negative experiences with cadaver dissection indicating the need for improved support (Table 1).

In addition, simulation‐based training played a vital role in improving the management and assessment skills for diabetic foot ulcers among podiatry professionals and students by enhancing their confidence, competence, and collaborative practice [30, 34, 41]. In the realm of debridement, the studies by Jackson et al. [40] and Grollo et al. [29] highlight the effectiveness of realistic simulation tools and workshops in significantly boosting podiatry students' debridement skills and confidence, with Banwell et al. [35] demonstrating how 3D printed models can similarly enhance scalpel skills in foot ulcer management (Table 1).

## 4 DISCUSSION

The aim of this scoping review was to explore the scientific literature on the topic of podiatry simulation for teaching and learning, and to map the study aims, simulation modalities and purposes, and the study findings with the conducted study designs as well as identifying any research gaps for exploration in future studies. The findings of this scoping review, the first to our knowledge, highlight an overall low volume of studies, which is consistent in medical simulation, with a lack of high‐level trials and a need for further investigation [51, 52, 53, 54]. Content analysis revealed a variety of simulation topics, which included: skills, communication and professionalism, clinical competencies and patient safety, educational enhancement, and anatomy and histology education.

This scoping review revealed a diverse utilization of simulation modalities, ranging from low‐fidelity inanimate objects such as fruit, to high‐fidelity simulations and VR. Previous research indicates mixed effectiveness between low‐ and high‐fidelity simulations in healthcare education, with high‐fidelity enhancing decision‐making and skills but not always translating to clinical practice [55, 56, 57], whereas low‐fidelity has been demonstrated as cost‐effective for gaining basic skills [55, 56]. Both types are valuable, influenced by the learner's stage, with low‐fidelity suitable for beginners and high‐fidelity for more advanced students [58, 59, 60, 61, 62]. However, the effectiveness of these simulations relies less on fidelity and more on well‐designed clinical scenarios, appropriate debriefing, and effective feedback, which are crucial for enhancing learning outcomes regardless of the simulation's complexity [22, 63, 64].

The results of the current study showed that simulation‐based training in podiatry has potential to enhance assessment, management and debridement skills for diabetes‐related foot ulcers, boosting confidence, competence, and collaborative practice through realistic tools and workshops including the use of 3D printed foot models. Podiatrists plays a vital role in diabetes‐related foot ulcer management through regular conservative sharp debridement, essential for reducing peak plantar pressures, reducing bioburden and serving as a fundamental prevention strategy against complications [65, 66]. The review's findings underscore the importance of integrating simulation into podiatry curricula to bridge the gap between theoretical knowledge and practical application particularly in the critical area of diabetes‐related foot care [29, 41]. Furthermore, the use of innovative simulation technologies, such as 3D printed models, not only provides more authentic scenarios but also offers a safe environment for students to practice and refine their skills [29, 35]. This approach underscores the fundamental advantage of simulation in providing a risk‐free environment where learners can make mistakes, learn from them, and improve their competencies without endangering patient safety [1, 2]. In addition to simulation, audit tools such as the Podiatric Audit of Surgery and Clinical Outcome Measurement (PASCOM‐10) can play a crucial complementary role in podiatric education and practice by providing robust clinical data that helps track patient outcomes and procedural success rates [67]. This data‐driven approach can be used alongside simulation to create a more comprehensive training environment, where simulation helps to refine procedural skills and PASCOM‐10 aids in assessing real‐world clinical impact, thus encouraging evidence‐based learning and continuous professional development.

The review identified diverse simulation modalities in podiatry education but highlighted a lack of standardized protocols, underscoring the need for research to establish best practices for enhancing education quality and efficiency [68, 69, 70]. The results indicate that there is a critical gap in high‐quality comparative research to evaluate the effectiveness of various simulation technologies and methodologies, including high‐fidelity versus low‐fidelity simulations. Therefore, comprehensive studies are essential to guide evidence‐based decisions on incorporating simulation into podiatry curricula, optimizing educational resources, and improving student learning outcomes [71].

Additionally, the existing simulation research in podiatry education primarily covers basic manual clinical skills, with a notable deficiency in studies on advanced procedures and interprofessional education [72, 73]. There is a critical need for expanding research to include simulations of complex procedures and collaborative scenarios that reflect the dynamics of clinical healthcare settings [74]. Addressing this gap by integrating advanced simulations requiring cross‐disciplinary teamwork is crucial for adequately preparing students for the multifaceted challenges of clinical practice [74].

Furthermore, the limited presence of randomized controlled trials (RCTs) in podiatry simulation research highlights a significant methodological gap emphasizing the necessity for more rigorous study designs to solidify the evidence base [75, 76, 77]. Implementing RCTs is essential for enhancing evidence quality and deriving conclusive insights into the efficacy of various simulation methods in podiatry education [78]. Furthermore, there is a notable lack of research linking simulation training in podiatry to improved patient outcomes highlights another significant gap, with only one study [38] directly assessing the impact of simulation‐acquired skills on real patient care. Addressing this gap with studies that correlate simulation training with clinical performance and patient outcomes is crucial for validating simulation's role in enhancing healthcare delivery [79, 80].

Future research in podiatry simulation should address the lack of longitudinal studies on its long‐term impact on clinical performance, patient outcomes, and career progression. There is a need for comparative effectiveness research to assess the benefits of different simulation modalities including high‐fidelity versus low‐fidelity simulations and VR. Research is also needed to link simulation training directly to improved patient outcomes and identify effective simulation techniques and fidelity levels for podiatry education. Additionally, establishing standardized approaches and best practices for simulation in podiatry is essential. Expanding research to include advanced podiatric procedures and interprofessional collaboration will better prepare students for clinical practice.

This present review has several limitations. The search for literature yielded 21 entries that met the inclusion standards. Limiting the search to only English‐language publications may have impacted the quantity of studies included potentially overlooking relevant research conducted in other languages. Considerable differences in how the included studies assessed and reported their parameters made it difficult to aggregate the findings. Given that nearly all the selected studies originated from high‐income nations with advanced podiatry education systems, the generalizability of these outcomes to contexts beyond these specific settings is limited.

## 5 CONCLUSION

The scoping review identified 21 studies assessing podiatry simulation in teaching and learning settings, which were focused on skill improvement, communication and professionalism, clinical competencies and patient safety, educational enhancement, and anatomy and histology education. The results highlight the limited research on simulation modalities in podiatry education, pointing out the need for longitudinal studies and research on the comparative effectiveness of different simulation technologies. It reveals a focus on short‐term outcomes without substantial evidence on long‐term impacts on clinical practice and patient care emphasizing a significant research gap. To advance podiatry education's relevance to clinical outcomes and patient health, there is a call for future studies to standardize simulation practices, investigate the enduring benefits of simulation training, and assess the effectiveness of various simulation tools.

## AUTHOR CONTRIBUTION

**Naomi Anning**: Conceptualization; Methodology; Data curation; Formal analysis; writing ‐ original draft; Writing ‐ review & editing. **Peta Tehan**: Methodology; Data curation; Formal analysis; Writing ‐ review & editing; Validation; Supervision.

## CONFLICT OF INTEREST STATEMENT

The authors declare that there are no conflicts or competing interest.

## ETHICS STATEMENT

Not applicable.

## RIGHTS AND PERMISSIONS

**Open Access:** This article is licensed under a Creative Commons Attribution 4.0 International License, which permits use, sharing, adaptation, distribution, and reproduction in any medium or format, as long as you give appropriate credit to the original author(s) and the source, provide a link to the Creative Commons license, and indicate if changes were made. The images or other third‐party material in this article are included in the article's Creative Commons license, unless indicated otherwise in a credit line to the material. If material is not included in the article's Creative Commons license and your intended use is not permitted by statutory regulation or exceeds the permitted use, you will need to obtain permission directly from the copyright holder.

## Data Availability

Deidentified data are held securely with the lead author and may be available upon request.
